# Global forecasting of thermal health hazards: the skill of probabilistic predictions of the Universal Thermal Climate Index (UTCI)

**DOI:** 10.1007/s00484-014-0843-3

**Published:** 2014-05-25

**Authors:** F. Pappenberger, G. Jendritzky, H. Staiger, E. Dutra, F. Di Giuseppe, D. S. Richardson, H. L. Cloke

**Affiliations:** 1 Forecast Department, European Centre for Medium-Range Weather Forecasts, Reading, UK; 2 University of Freiburg, Freiburg, Germany; 3 University of Reading, Reading, UK

**Keywords:** Universal Thermal Climate Index (UTCI), Ensemble forecasting, Temperature forecasting, Thermal comfort, Thermal stress, NWP

## Abstract

Although over a hundred thermal indices can be used for assessing thermal health hazards, many ignore the human heat budget, physiology and clothing. The Universal Thermal Climate Index (UTCI) addresses these shortcomings by using an advanced thermo-physiological model. This paper assesses the potential of using the UTCI for forecasting thermal health hazards. Traditionally, such hazard forecasting has had two further limitations: it has been narrowly focused on a particular region or nation and has relied on the use of single ‘deterministic’ forecasts. Here, the UTCI is computed on a global scale, which is essential for international health-hazard warnings and disaster preparedness, and it is provided as a probabilistic forecast. It is shown that probabilistic UTCI forecasts are superior in skill to deterministic forecasts and that despite global variations, the UTCI forecast is skilful for lead times up to 10 days. The paper also demonstrates the utility of probabilistic UTCI forecasts on the example of the 2010 heat wave in Russia.

## Introduction

The extreme heat wave that affected Russia in June 2010 killed over 55,000 people. In China, extreme winter conditions in January 2008 affected 77 million people, leading to costs of over 21 billion USD (Em-Dat 2014). During the hot summer of 2003 in western and southern parts of Europe, 55,000 deaths were attributed as heat related (Koppe et al. 2004), 35,000 of which were during the hottest period in early August. An early understanding of the thermal health hazards associated with extreme heat or cold can serve to minimize the impact by increasing preparedness in the affected region. For this reason forecasting of thermal indices is routinely carried out by national weather services (Staiger et al. 1997) usually based on high-resolution numerical weather prediction (NWP) forecasts.

In order to determine thermal stress, several factors, including air temperature, wind velocity, water vapour pressure, short- and long-wave radiant fluxes, physiological strain, behaviour and the autonomous human thermoregulatory system, need to be considered (Havenith 2001; Jendritzky et al. 2009; Parsons 2003). There are more than 100 indices used to assess thermal health hazards. The first indices to be widely used were based on a simple two-parameter combination of air temperature and humidity for ‘warm’ indices and air temperature and wind speed (wind chill) for ‘cold’ indices (Blazejczyk et al. 2012). Over the last 30–40 years, a second generation of still relatively simple human heat budget models have been developed which consider the core and the shell of the human body known as 2-node (core/shell of the human body), and these have improved the assessment of the thermal environment. Examples are the ‘physiological equivalent temperature PET’ (Hoeppe 1999; VDI 2008), and OUT-SET* (Pickup and de Dear 2000), with further examples described in Blazejczyk et al. (2012). One of the earliest simple heat budget models was the Klima-Michel model developed by the German national weather service, Deutscher Wetterdienst (DWD) (Jendritzky et al. 1979), which translated Fanger’s (1970) predicted mean vote (PMV) equation to outdoor conditions. This was mainly achieved by writing a radiation scheme in order to calculate the mean radiant temperature *T*_*mrt*_ (by which short- and long-wave radiant fluxes refer to an upright standing human being) and was based on easily available meteorological data. The outcome is ‘perceived temperature *PT*’ that relates in its development to an improved radiation scheme and the 2-node thermo-physiology outside of the PMV comfort region (Staiger et al. 1997; VDI 2008; Staiger et al. 2012). The radiation scheme for calculating *T*_*mrt*_ based on NWP meteorological data in our paper is currently used by the DWD.

To calculate the entire heat exchange between the human body and its environment, 2-m air temperature (Ta), wind velocity at body height derived from 10-m wind speed (*v*), 2-m water vapour pressure (*e*) and *T*_*mrt*_ are needed as meteorological input variables. The limitations of the two-parameter indices are obvious because they do not consider the human heat budget and hence ignore issues such as physiology and clothing. Although the 2-node heat budget models represent an improvement over the simple indices, they still make several simplifications considering thermo-physiology and heat exchange theory (for example, the effects of clothing). To exploit more recent scientific developments and to minimize the various shortcomings of the former assessment procedures (Jendritzky et al. 2012), the Universal Thermal Climate Index (UTCI) was developed using one of the most advanced and comprehensively validated (Psikuta et al. 2012) multi-node models of human heat transfer and thermo-regulation (Fiala et al. 2012; Fiala et al. 2001). The UTCI development was performed by a multidisciplinary expert team in the framework of a commission of the International Society of Biometeorology (ISB) and of COST Action 730 (Jendritzky et al. 2009) under the ‘umbrella’ of the World Meteorological Organisation Commission for Climatology (WMO-CCl). The UTCI can be applied to key applications in human biometeorology, such as daily forecasting and warnings, urban and regional planning, environmental epidemiology and climate impact research; it is applicable for all climates.

Forecasts of thermal indices have to rely on NWP models to provide the required meteorological input at future times. The quality of the thermal index forecast will be dependent on the quality of the meteorological forcing as well as on the definition of the index itself. Forecasts of thermal indices are usually made using single (deterministic) runs of NWP models. However, recent advances in NWP indicate that ensemble prediction systems (EPS) have higher skill than deterministic forecasts in forecasting meteorological variables over the medium term of 3 to 10 days (Bartholmes et al. 2009; Pappenberger et al. 2011b; Richardson 2000; Roulin 2007). Ensembles account for the unavoidable uncertainties in weather forecasting by providing multiple future weather scenarios, allowing the forecast to be expressed in terms of probabilities. This probabilistic information allows assessment of the most likely and extreme scenarios, facilitating better preparedness for any stakeholders involved (Pitt 2008). In addition, the potential costs and losses of precautionary actions, such as early warning provision to fuel suppliers, water resources and health care providers can be carefully assessed (Richardson 2000).

The UTCI was designed to be applicable in all climate regions, and global NWP ensembles can be used to forecast the UTCI anywhere in the world. However, before such forecasts can be used, it is essential to assess their skill over the area of interest. For a comprehensive evaluation, observations must be available on a global scale and be comparable in terms of error structure to the forecasts. Whilst direct observational measurements are inhomogeneously distributed globally (very sparse in some regions) and need careful quality control, recently developed reanalyses provide consistent, quality-controlled historical analysis of the state-of-the-global atmosphere based on a wealth of ground, atmospheric and satellite observational data. Here, the ERA-Interim reanalysis (Dee et al. 2011) is used as a global observation proxy.

Global ensemble forecasts from the integrated forecasting system (IFS) of the European Centre for Medium-Range Weather Forecasts (ECMWF) are used to provide the meteorological input required for the UTCI. The skill of the UTCI forecasts will depend on the quality of this meteorological forcing. The meteorological performance of the ECMWF ensemble forecasts is evaluated in detail elsewhere (Hagedorn et al. 2008, 2012; Hamill et al. 2008; Pinson and Hagedorn 2012; Richardson et al. 2013), and only a brief discussion is included here.

This paper provides an assessment of the UTCI using probabilistic NWP forcing on a global scale, demonstrating that the UTCI can be readily combined with forecast data. The objectives of this paper are to analyze the global behaviour of the UTCI using ECMWF reanalysis data and to assess the predictability of the UTCI using medium-range (10-day) probabilistic forecasts. The added value of these global probabilistic UTCI predictions will be assessed. This evaluation, using ECMWF ensembles as the meteorological forcing, provides a benchmark against which other forecasting systems can be compared.

## Methodology

The UTCI is calculated globally using meteorological input from ECMWFs high-resolution and ensemble forecasts. The quality of these forecasts is assessed by comparing the predicted UTCI against analyzed values computed using reanalysis data (as a proxy for the truth, in the absence of global observational data). Both deterministic and probabilistic evaluation scores are used.

## The Universal Thermal Climate Index (UTCI)

The UTCI is the result of an approach which developed more than a decade ago in the International Society of Biometeorology (ISB) Commission 6 and was later reinforced by COST Action 730 (Jendritzky et al. 2009). The various aspects of UTCI are comprehensively described in the final report of COST Action 730 (Jendritzky et al. 2009) and by 10 papers in a UTCI special issue on of Int J Biometeorol (56; 2012). The development pooled the resources of multidisciplinary experts in the fields of thermo-physiology, biology, mathematical modelling, occupational and environmental medicine, clothing research and meteorological data handling.

The UTCI is based on Fiala et al.’s (2012, 2001) advanced multi-node model of thermo-regulation. Thermo-regulation is the ability of an organism to keep its body temperature within certain boundaries, even when the surrounding temperature is very different (Eq. 1).

1 $$ UTCI\sim f\left(\mathrm{Ta}, Tmrt,v,e\right) $$

**Fig. 1:**
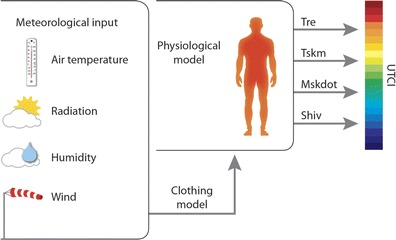
Concept of the UTCI derived from the dynamic multivariate response of the thermo physical UTCI-Fiala model (Fiala et al. 2012), which was coupled with a clothing model (Havenith et al. 2012). *Tre* rectal temperature, *Tskm* mean skin temperature, *Mskdot* sweat production, *Shiv* heat generated by shivering. Figure after Błażejczyk et al. 2013

Fiala’s model is coupled with a state-of-the-art clothing model (Havenith et al. 2012) that takes into consideration Ta-driven behavioural adaptation of clothing insulation of the general public (see Fig. 1). The UTCI has been derived conceptually as an equivalent temperature. Thus, for any combination of *Ta*, *T*_*mrt*_, *v*, and e v, i.e. the thermal stress of the actual environment, UTCI is defined as that air temperature that would elicit the same dynamic physiological response (strain) under a set of reference conditions. The reference conditions are defined as follows: walking at a speed of 4 km/h (which is equivalent to a metabolic rate of 2.3 MET or 135 W/m^2^), *T*_*mrt*_ equal to UTCI, a 10-m wind speed of 0.5 m/s and relative humidity of 50 % that is capped at a water vapour pressure of 20 hPa for Ta > 29 °C. The thermal stress assessment scale (see Table 1) has been derived from the modelled physiological and psychological response (Broede et al. 2012).

**Table 1:** UTCI equivalent temperature and stress category (Broede et al. 2012)

UTCI (C) range	>46	38–46	32–38	26–32	9–26	0–9	0 to −13	−13 to −27	−27 to −40	<−40
Stress category	Extreme heat stress	Very strong heat stress	Strong heat stress	Moderate heat stress	No thermal stress^a^	Slight cold stress	Moderate cold stress	Strong cold stress	Very strong cold stress	Extreme cold stress

^a^Thermal comfort zone, which provides a subjective satisfaction with the thermal environment defined for UTCI values between 18 and 26 °C

A state-of-the-art adaptive clothing model is integrated into the UTCI. This takes account of behavioural adaptation of clothing insulation for the general urban population and reduction of thermal and evaporative clothing resistance by wind and limb movements of the wearer. However, due to the lack of a reliable global database, the clothing model currently focuses on Western-style clothing. Hence, an improvement on the regional scale could be imagined by using data derived for local (traditional) clothes. Whilst the basic thermo-regulation of human beings is the same all over the world, there are differences in the sensitivity of local populations to thermal stress due to behavioural and physiological acclimatization. For indoor comfort, De Dear and Brager (2002) presented an ‘adaptive model of thermal comfort’. Unfortunately, for outdoor conditions, there is no generally accepted objective procedure available which explains all factors determining the level of adaptation on the population or individual level in all regions and scales. With respect to this issue, a meaningful interpretation of the standard thresholds to be tailored to the local experience is required when using UTCI (as is also true for any other indices). However, Koppe and Jendritzky (2005) suggested a possibly suitable approach which adjusts the thresholds of the thermal stress categories with respect to the thermal conditions that happened in the past couple of days to weeks.

Calculating the UTCI equivalent temperature by repeatedly running the original Fiala multi-node model is computationally intensive and thus too slow to be used for operational numerical weather forecasts and climate simulations. Therefore, a fast calculation using a polynomial approximation procedure has been developed and made available for open access (Broede et al. 2012). The numerical code to calculate *T*_*mrt*_ with NWP data was developed by Staiger (pers. comm.) and is provided to the authors courtesy of the DWD.

## The ECMWF forecasting system

### ECMWF forecasts

Here, ECMWF’s 10-day high-resolution forecasts (HRES) and 15-day ensemble forecasts (ENS) (Richardson et al. 2013) are used to provide the meteorological input to the UTCI predictions. The ENS takes into account the forecast uncertainty and consists of a control run and 50 perturbed simulations. These ensemble members are generated by a combination of perturbations in the initial conditions of the forecast and perturbations during the model integration to account for uncertainties in the model equations. The spread among the ensemble members is a measure of the confidence in the prediction. The first 10 days of the ENS are performed at a spatial resolution of approximately 32 km × 32 km forced by persisted sea surface temperature (SST) anomalies (updated every 24 h). After day 10, the model is coupled to the ocean model and has a spatial resolution of roughly 64 × 64 km. The 10-day high-resolution forecast uses the same model version as the ENS system, but runs are performed at a much higher horizontal (roughly 16 × 16 km) and vertical resolution.

### ECMWF reanalysis: observation proxy

Reanalysis involves reprocessing observational data spanning an extended historical period, incorporating a very large number of ground-based, ocean-, atmosphere- and satellite-based observations. A data assimilation system is used to transform these millions of observations into the model space to produce a dataset that can be regarded as a proxy for observations but with the advantage of providing spatio-temporal resolution unobtainable with a normal observational network. It should be noted that reanalyses, although constrained by the observations and data assimilation system, may suffer from effects of model errors; these impacts are discussed in the documentation of the reanalysis datasets (Dee et al. 2011). The latest global atmospheric reanalysis produced by ECMWF is ERA-Interim (ERA-I), which extends from 1 January 1979 to the present date (Dee et al. 2011). Gridded data products include a large variety of 3-hourly surface parameters, describing weather as well as ocean-wave and land-surface conditions, and 6-hourly upper-air parameters covering the troposphere and stratosphere. In this study, we use ERA-Interim as a proxy for global observations to generate an analysis of UTCI which will be used as a benchmark for forecast skill calculations.

To evaluate the skill of UTCI forecasts, 4 years of data were processed. A UTCI forecast was computed every day (with a lead time of 10 days) from 1 January 2009 to 31 December 2012 using both the high-resolution and 51-member ENS forecast.

### Evaluation scores

A set of well-established skill scores is used to assess the skill of the UTCI predictions. Deterministic forecasts from both HRES and ENS were evaluated using the anomaly correlation coefficient (ACC). The Brier skill score (BSS) and the continuous rank probability skill score (CRPSS) were used to evaluate the ENS probabilistic forecasts.

### Anomaly correlation coefficient (ACC)

The anomaly correlation coefficient is a measure of the similarity between two signals or patterns (ignoring any potential offsets or biases). Both forecasts and observations are first expressed as anomalies from climatology before computing the correlation between them. This minimizes the seasonal effect (Stevenson 2006).

2 $$ ACC=\frac{\sum_{i=1}^m\left(\widehat{x_i^{\hbox{'}}}-\overset{-}{\widehat{x\hbox{'}}}\right)\left({x}_i^{\hbox{'}}-\overline{x\hbox{'}}\right)}{{ Ms}_{\widehat{x\hbox{'}}}{s}_{x^i}} $$

*x*_*i*_^'^ and $\hat{x_i^{\hbox{'}}}$ are the observed and forecast anomalies, respectively. ${s}_{\hat{x^{\hbox{'}}}}$ and ${s}_{x^i}$ are the standard deviations of the anomalies. *M* being the number of cells and the overbar expressing the mean.

The higher the anomaly correlation, the better is the performance of a forecast system. The ACC, whilst a good measure of forecast skill, is not sensitive to bias, and hence, a good correlation should not be used in isolation to assess a forecast if bias is important. The ACC is used to assess deterministic forecast skill. However, it does not provide information on the range of possibilities or uncertainty in the forecast, which is provided by an ensemble of forecasts. Two probabilistic scores are therefore also used; the continuous rank probability skill score (CRPSS) (Hersbach 2000) and the Brier skill score (BSS) (Murphy 1973).

### Brier skill score (BSS)

The Brier score measures the mean squared probability error for binary events (e.g. UTCI greater than 32 °C, see Eq. 3). The climatological probability of the event can be considered as a no-skill reference forecast. The Brier skill score measures the improvement of the ECMWF forecasts with respect to this reference. The Brier skill score has a maximum of 1 (indicating a perfect deterministic forecast; Murphy 1973), whilst positive values indicate higher skill than the climate benchmark.

3 $$ BSS=1-\frac{\frac{1}{n}{\displaystyle {\sum}_{t=1}^n}{\left(\hat{p_t}-{y}_t\right)}^2}{\frac{1}{n}{\displaystyle {\sum}_{t=1}^n}{\left(c-{y}_t\right)}^2} $$

BSS: Brier skill score
$$ \hat{p_t} $$
Probability assigned to the event by the *t* ^th^ forecast
*y*_*t*_: Equals 1 if the *t* ^th^ observation corresponds to an event, 0 otherwise
*c*: Climatological probability of the event (here, based on a 30-year record)
*n*: Number of cases

### Continuous rank probability skill score (CRPSS)

The continuous rank probability score (CRPS) is calculated as the square differences in the cumulative probability space between a probabilistic forecast and observation (see Eq. 4). It is transformed into a skill score (CRPSS) by comparing it to a climatological forecast based on a 30-year record. Seasonal means derived from the reanalysis data are used to provide the reference climate. The higher the CRPSS, the better the forecast, with a maximum value of 1 and positive values indicating skill with respect to the climate benchmark.

4 $$ CRPS={\int}_{-\infty}^{\infty}\left[P(x)-H\left(x-{x}_a\right)\right] dx $$

where *x* is the forecast variable, *x*_*a*_ is the observed value, *P(x)* is the cumulative distribution function of *x* and *H*(*x* − *x*_*α*_) is the Heaviside function which is 0 when (*x* − *x*_*α*_) < 0 and 1 otherwise.

## Results

### Performance of ECMWF NWP forecasts

**Fig. 2:**
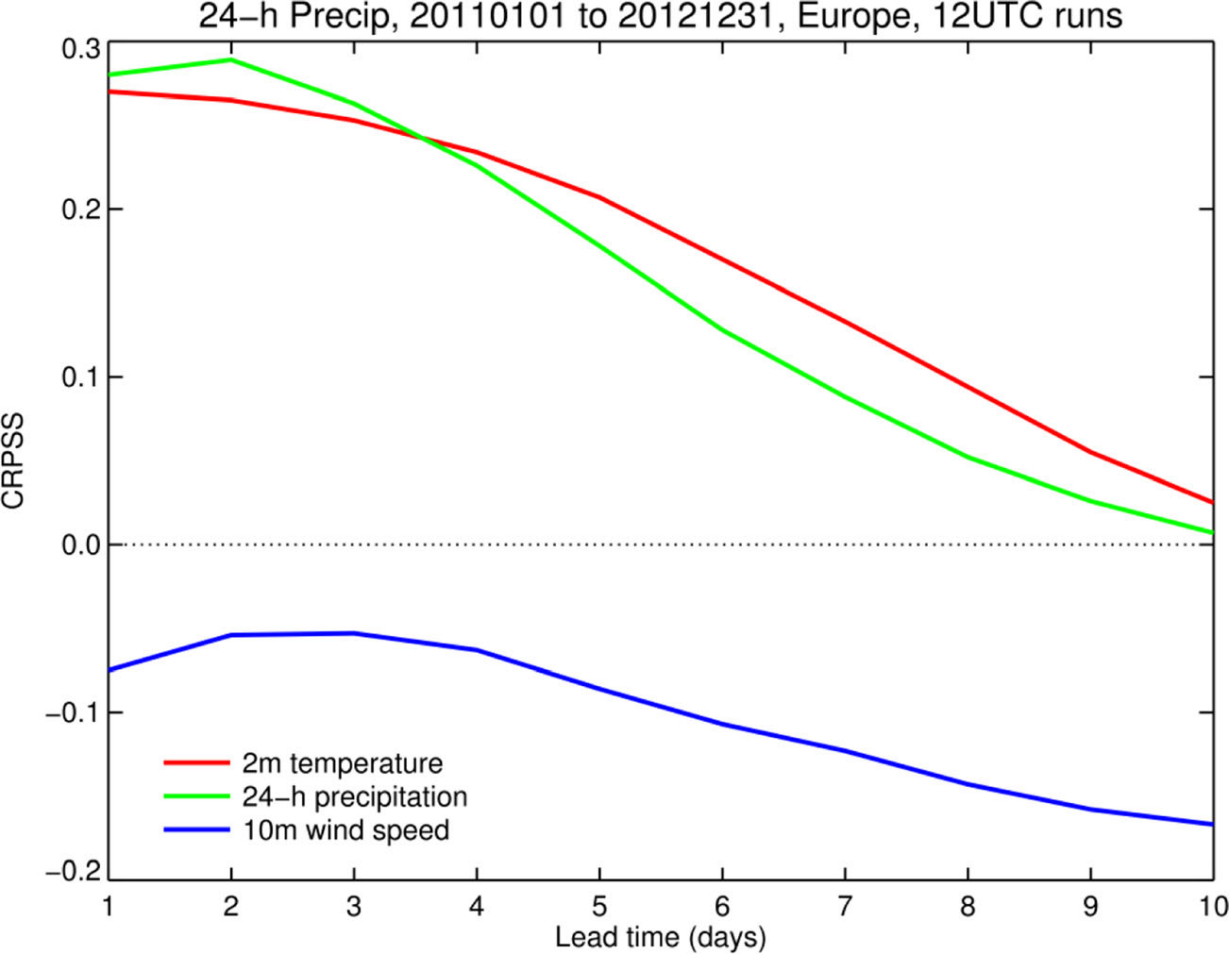
CRPSS for 2011–2012 at 12UTC in Europe as a function of lead time for precipitation, 2-m temperature and 10-m wind speed

Before considering the skill of the UTCI predictions, we briefly consider the forecast skill of the meteorological parameters used as input to the UTCI calculations. The performance of the ECMWF forecasting system is published in annual reports (Richardson et al. 2013) and regularly updated in the ECMWF Newsletters and on the ECMWF web site (http://www.ecmwf.int). As an example, Fig. 2 shows the CRPSS for 2011–2012 at 12UTC in Europe as a function of lead time for ENS forecasts of precipitation, 2-m temperature and 10-m wind speed (three of the key variables used in the UTCI calculation, verification against ground observations, SYNOP stations). The CRPSS decreases with increasing lead time indicating that skill decreases relative to the climate reference. The skill is clearly positive for temperature and precipitation and negative for wind speed. The low CRPSS for 10-m wind speed compared to 2-m temperature or precipitation is due to systematic errors in the wind speed forecast, to which the CRPS is sensitive; for a further discussion on wind forecast performance, see Pinson and Hagedorn (2012). This negative skill indicates that the performance of the wind forecast could negatively affect the performance of the UTCI forecast. However, the UTCI is a non-linear combination of all these variables, and the effect of the wind error on the skill of the final UTCI product cannot be predicted in advance.

Statistical post-processing of the ensemble forecast can, in principle, correct systematic errors in the meteorological parameters used by the UTCI. Indeed, studies have demonstrated substantial improvements in ECMWF probabilistic forecasts for 10-m wind (Courtney et al. 2013) as well as for 2-m temperature (Hagedorn et al. 2008, 2012) and precipitation (Hamill et al. 2008). However, in the present study, the ECMWF forecast data is used directly with no attempt to account for systematic errors in the model. The skill of the resulting UTCI forecasts can therefore be considered as a lower bound to what may be achievable with appropriate calibration.

### Global UTCI climatology

**Fig. 3:**
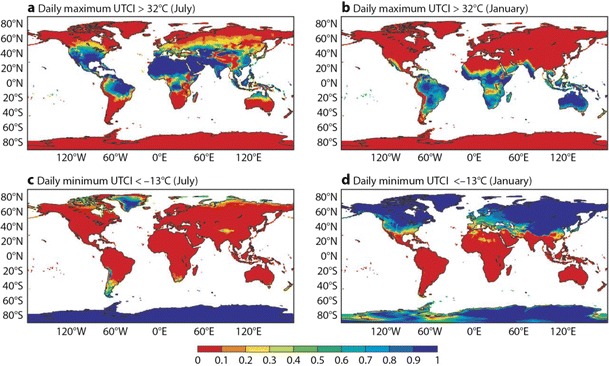
**a** Fraction of days of the UTCI (daily maximum) greater than 32 °C in July; **b** fraction of days of the UTCI (daily maximum) greater than 32 °C in January; **c** percentage of days of the UTCI (daily minimum) lower than −13 °C in July; **d** percentage of days of the UTCI (daily minimum) lower than −13 °C in January

The benchmark used for the evaluation of the UTCI forecasts is a 30-year climatology of heat stress and cold stress across the globe derived using the ERA-I reanalysis. For 30 years of data, Fig. 3a, b shows the percentage of days in which the daily maximum UTCI exceeds 32 °C for January and July, respectively. This UTCI value marks the boundary for strong heat stress (see Table 1). A UTCI of −13 °C indicates the boundary for strong cold stress (see Table 1), and the percentage of days with UTCI below this threshold are displayed in Fig. 3c, d. Figure 3a, b uses the daily maximum UTCI, whereas Fig. 3c, d is calculated using the daily minimum UTCI. As expected, the index shows a clear seasonal pattern. Strong heat stress affects almost all southern hemisphere land areas in January, northern hemisphere (especially equatorwards of 40 N) in July and tropical regions in both months. In addition, there are significant regional variations, mainly related to orography. Strong cold stress mainly affects the winter-time northern hemisphere (Fig. 3c, d), with notable longitudinal gradients across Europe and western North America, consistent with the climatological temperature patterns. However, it should be noted that there is not a 1:1 correspondence with temperature: as expected, the UTCI is providing additional information (as discussed in the next section). Some regions are subject to stress over 90 % of days in these months, for example, large parts of Australia are under permanent strong heat stress in January, whereas large parts of Asia and North America are under almost constant strong cold stress in January.

### Sensitivity to NWP forecast variables

**Fig. 4:**
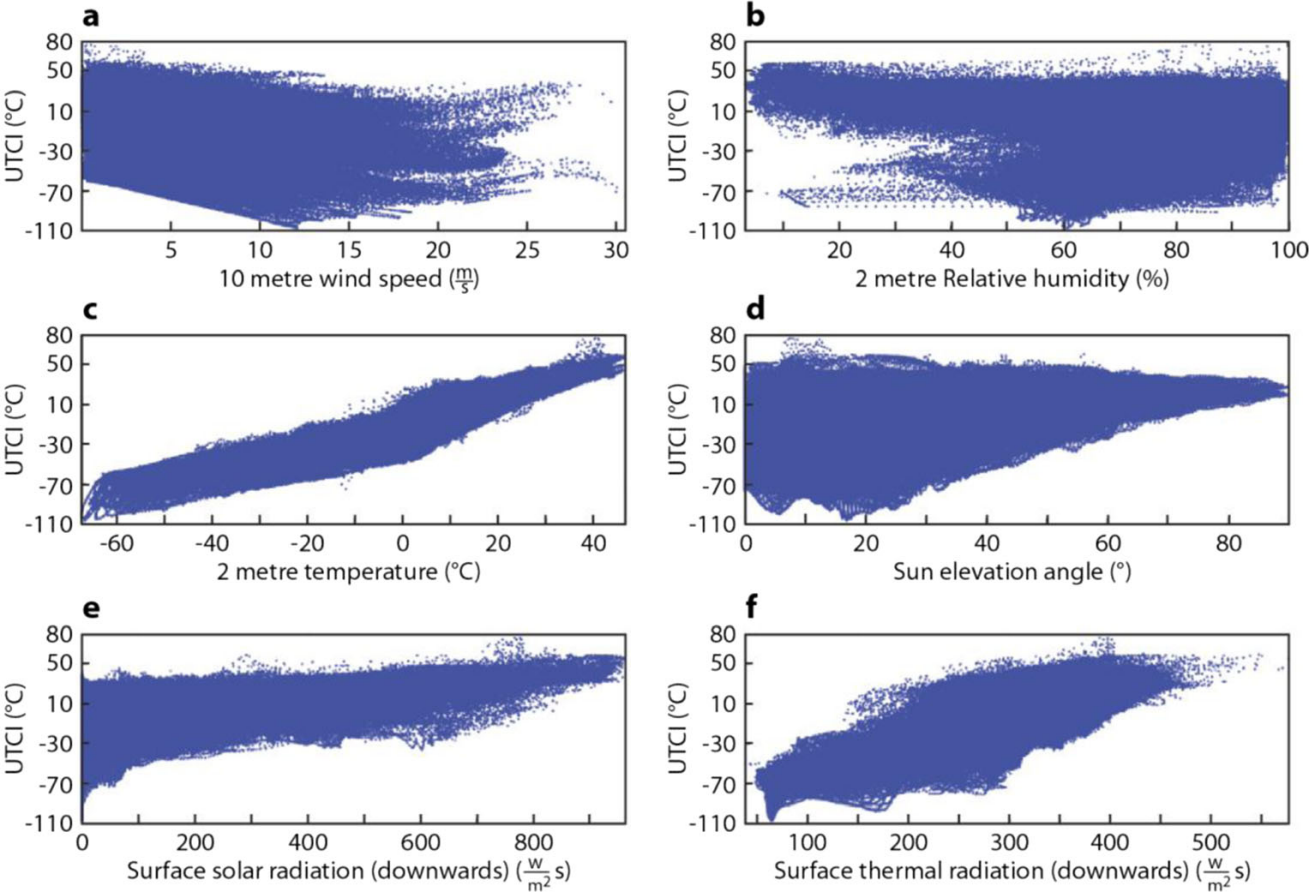
Plot of meteorological inputs against UTCI to illustrate the associated dependencies based on all grid points and all reanalysis data

In order to understand the relationship between the NWP forecast variables used to generate the UTCI and the UTCI values themselves, sensitivity plots were constructed (Fig. 4). These show the relationship between computed UTCI values and each meteorological input variable for the entire reanalysis period and all grid points. Although the UTCI has some linear dependencies on air temperature, a given temperature can lead to a wide range of UTCI values. This underlines the value of calculating the UTCI rather than relying only on temperature forecasts as an indicator of potential heat stress. The UTCI also shows sensitivity to wind, which shows a distinct lower boundary. The UTCI is more sensitive to wind than previous indices because it accounts for changes in clothing insulation and vapour resistance caused by wind and body movement (Havenith et al. 2012). Figure 4a shows a distinctive tailing behaviour at wind speeds of over 17 m/s; this is because of the polynomial approximation for the UTCI used in this study which has not been optimized beyond this range. A limit of a maximum wind speed of 17 m/s should thus be considered in the future. This is responsible for the extremely low UTCI values. The solar elevation angle clearly influences the lower bound for the UTCI, as do the solar and thermal radiation. These input variables are themselves correlated. This makes a full interpretation more difficult as higher order dependencies (dependencies on more than one input variable) cannot be determined from this one-dimensional analysis (Cloke et al. 2008).

### UTCI forecast

A UTCI forecast was calculated every day (with a lead time of 10 days) from 1 January 2009 to 31 December 2012 using both the HRES and 51-member ENS inputs. The skill is assessed for these 4 years of data using ACC, BSS and CRPSS.

### Similarity between forecast and observed UTCI

**Fig. 5:**
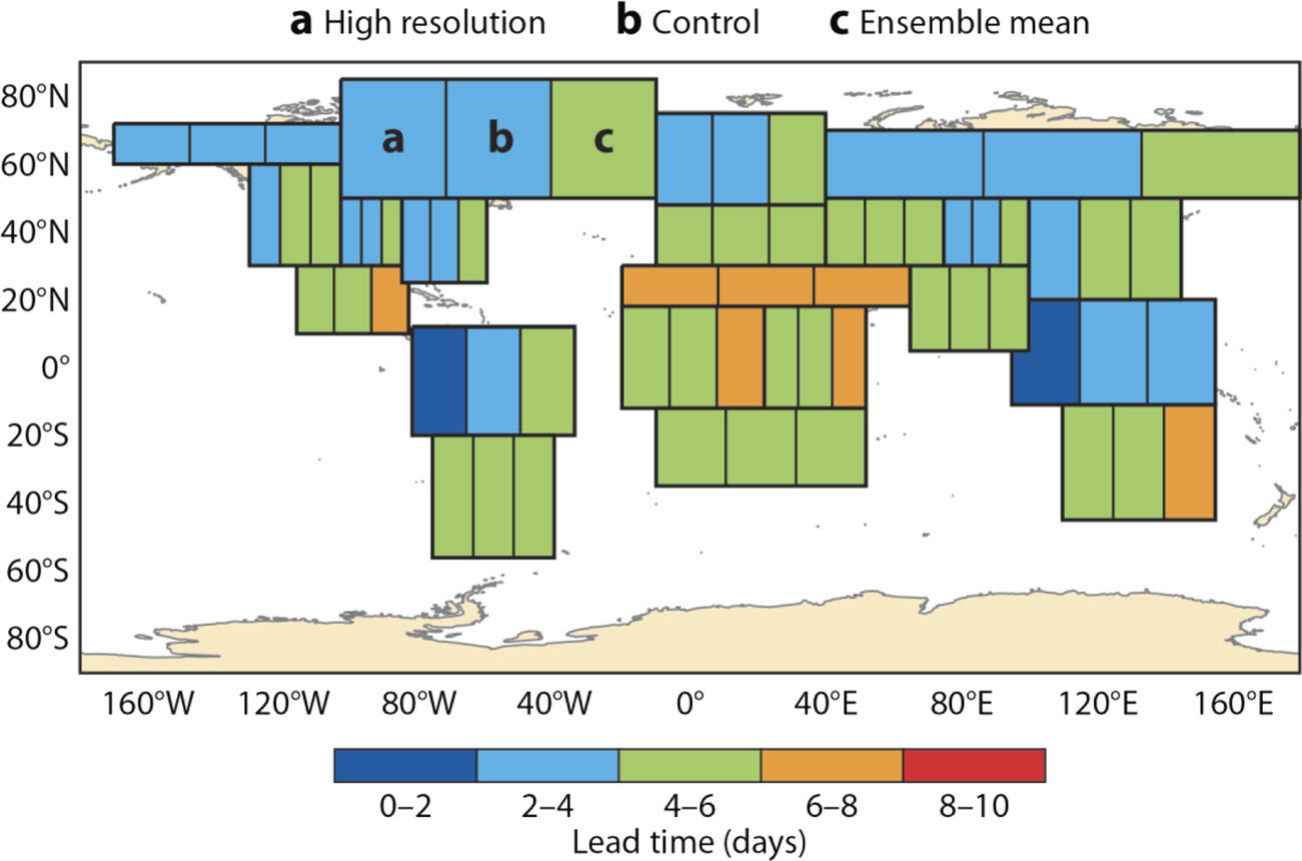
Maximum lead time at which anomaly correlation reaches 60 %. Each area is sub-divided into three boxes (*a*, *b*, *c*) as labelled on figure. The *left-hand box* represents the high-resolution forecast, the *middle box* the control forecast and the *right-hand box* the ensemble mean

**Fig. 6:**
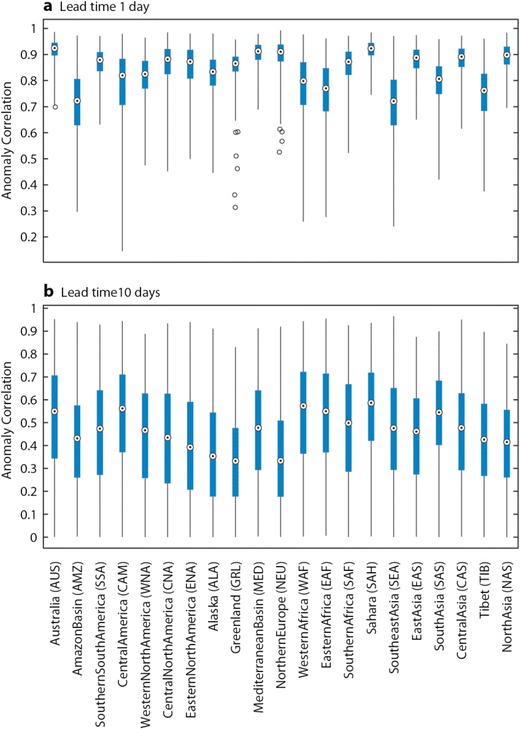
Anomaly correlation for ensemble mean forecasts of UTCI for different regions for lead times of 1 day (**a**) and 10 days (**b**) . The *box plots* show the uncertainty derived through bootstrapping. The *white circle* illustrates the mean whilst the *box* indicates the 25th and 75th percentile. The *whiskers* of the box plot extend to the 95th and 5th percentile. *Blue circles* indicate outliers

**Fig. 7:**
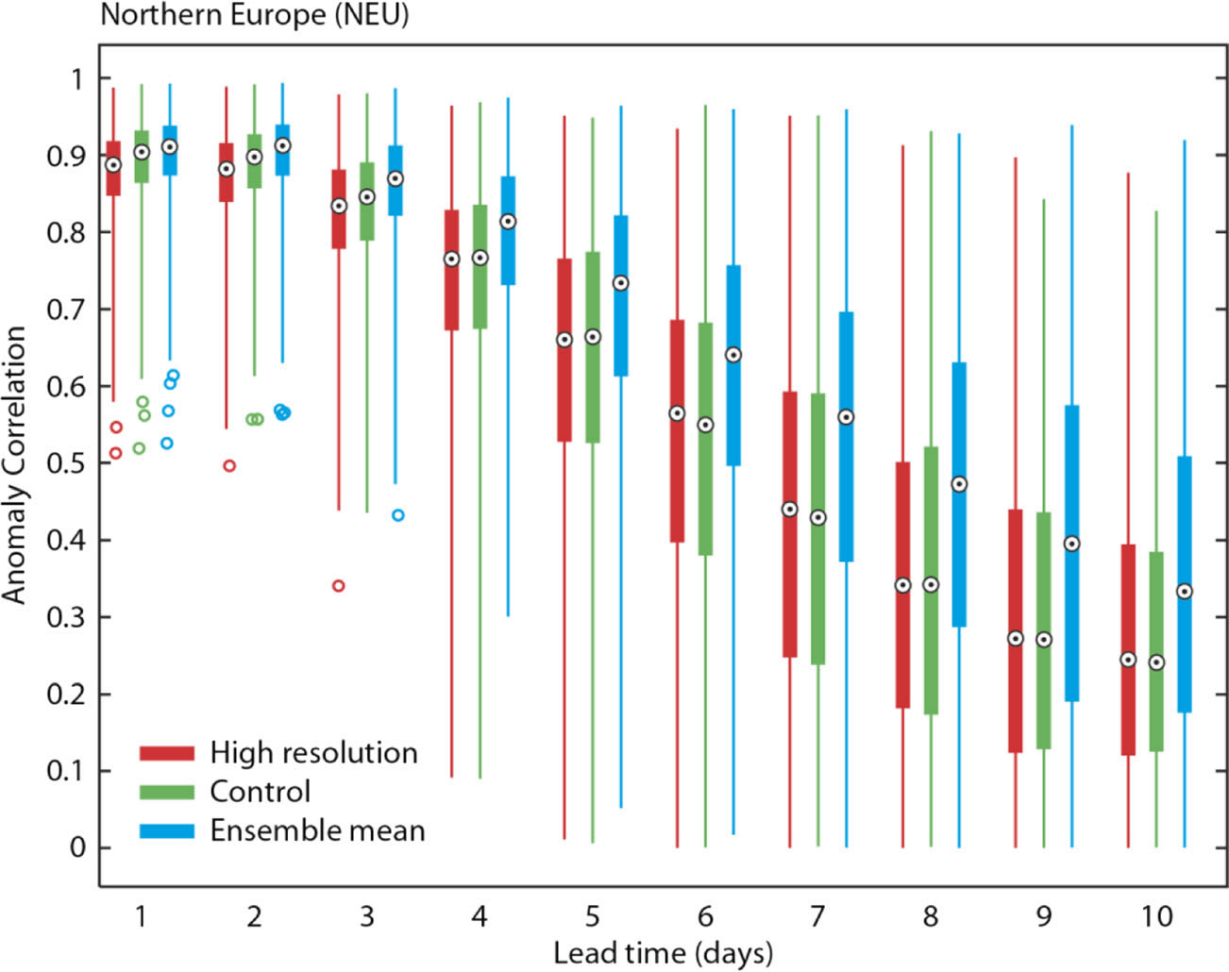
Anomaly correlation for the Northern European area and the three different UTCI forecasts. The *box plots* show the uncertainty derived through bootstrapping. The *circle* illustrates the mean whilst the *box* indicates the 25th and 75th percentile. The *whiskers* of the box plot extend to the 95th and 5th percentile. *Coloured circles* indicate outliers

The deterministic high-resolution, control and ensemble mean forecasts of UTCI are compared with observations using the ACC. In Fig. 5, the maximum lead time for which ACC is above 60 % is shown for a list of regions across the globe (see Table 2). The skill is shown for each of the three available forecasts: the left-hand box of each box indicates the skill of the high-resolution forecast, the middle box the skill of the control forecast and the right-hand box the skill of the ensemble mean. For example, the colour green indicates that the ACC drops below 60 % at a lead time of 4–6 days. The maximum predictability according to this measure is 6–8 days, with most regions achieving values of 4–6 days. In general, the ensemble mean displays higher predictability than the other two forecasts. The results in Fig. 5 are very encouraging in terms of forecast skill but give little indication about the uncertainty and spread of the ACC. The uncertainty is shown using box plots of the anomaly correlation for the different regions for lead times of 1 day (Fig. 6a) and 10 days (Fig. 6b) for the ensemble mean. The uncertainty is derived by bootstrapping the sample using 80 % of available data points. Uncertainty increases with lead time, and the different regions exhibit varying spread. For example, the Mediterranean Basin, Sahara and Northern Europe show comparatively small variation compared to Central America for day 1, which reflects the distribution also found through the verification of the meteorological forecasts (not shown). At the lead time of 10 days, none of the distributions for the different regions are significantly different. In Fig. 7, the ACC for the high-resolution, control and ensemble mean forecasts are plotted for the Northern European area for all lead times from 1 to 10 days. There is a clear drop of ACC with lead time, whilst the ensemble mean shows higher skill overall (although it is only a statistically significant difference in the lead times of day 1 and 2—not shown).

**Table 2:** List of regions used in this study adapted from Giorgi and Francisco (2000)

Name	Acronym	Latitude (°)	Longitude (°)
Australia	AUS	45 S–11 S	110 E–155 E
Amazon Basin	AMZ	20 S–12 N	82 W–34 W
Southern South America	SSA	56 S–20 S	76 W–40 W
Central America	CAM	10 N–30 N	116 W–83 W
Western North America	WNA	30 N–60 N	130 W–103 W
Central North America	CAN	30 N–50 N	103 W–85 W
Eastern North America	ENA	25 N–50 N	85 W–60 W
Alaska	ALA	60 N–72 N	170 W–103 W
Greenland	GRL	50 N–85 N	77 W–10 W
Mediterranean Basin	MED	30 N–48 N	10 W–40 E
Northern Europe	NEU	48 N–75 N	10 W–40 E
Western Africa	WAF	12 S–18 N	20 W–22 E
East Africa	EAF	12 S–18 N	22 E–52 E
Sahara	SAH	18 N–30 N	20 W–65 E
Southern Africa	SAF	35 S–12 S	10 W–52 E
Southeast Asia	SEA	11 S–20 N	95 E–155 E
East Asia	EAS	20 N–50 N	100 E–145 E
South Asia	SAS	5 N–30 N	65 E–100 E
Central Asia	CAS	30 N–50 N	40 E–75 E
Tibet	TIB	30 N–50 N	75 E–100 E
North Asia	NAS	50 N–70 N	40 E–180 E

### Skill in probabilistic prediction of cold and warm stress

**Fig. 8:**
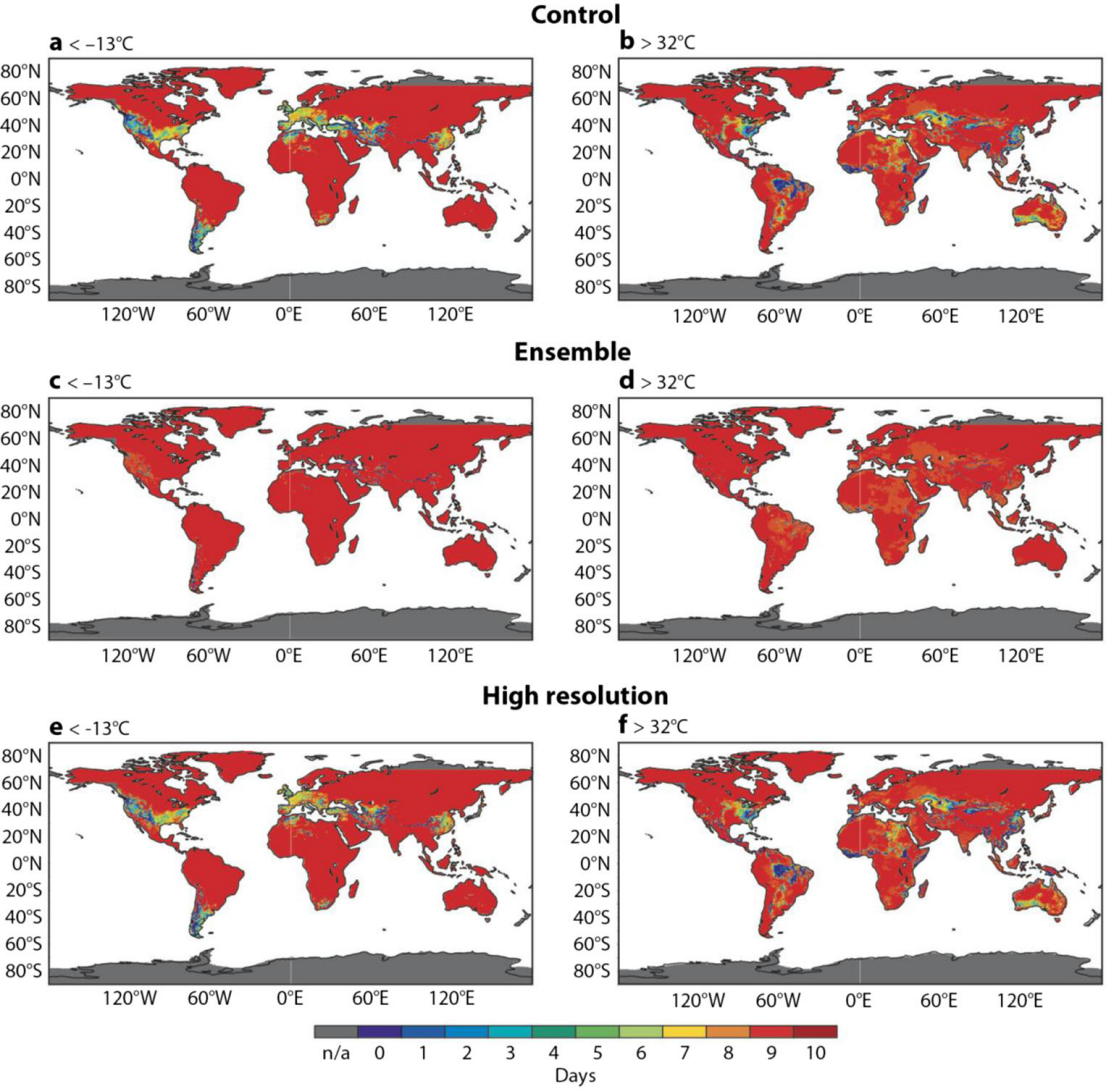
Maximum predictability until Brier skill score reaches zero (equivalent to no skill) **a** for the control forecast being above 32 °C, **b** for the control forecast being above 32 °C, **c** for the ensemble forecast being below −13 °C, **d** for ensemble forecast being above 32 °C, **e** for the high-resolution forecast being below −13 °C, **f** for the high-resolution forecast being above 32 °C. The *colour scale* indicates the maximum predictability meaning *dark red* stands for skilful predictability of 10 days

The previous analysis considered deterministic predictions of the UTCI in general. This section assesses the probabilistic information in the full ensemble distribution and focuses on the important ‘strong’ UTCI categories. The BSS for the thresholds given by strong heat stress (>32 °C) and strong cold stress (<−13 °C) are analyzed. When the Brier skill score drops below zero, there is no skill compared to using climate information. Figure 8 displays the lead time at which the zero skill threshold is crossed. For example, the BSS for the control forecast of the cold stress is shown in Fig. 8a. Large parts of the globe exhibit a predictability of 9 days, although the predictability is lower in central Europe, America, parts of China and South America. For the UTCI threshold of <−13 °C, particular mountainous areas (such as the Alps or Andes) have low skill values, probably due to the slightly different underlying topography of reanalysis, high resolution and control. This may be addressed through height correction but should be performed on a regional scale. The ensemble predictions have a higher skill than the control or high-resolution forecast for both strong cold and strong heat stress, confirming the benefit of using the probabilistic information in the ensemble. Overall predictability is lower for strong heat stress than for strong cold stress, but otherwise, spatial patterns are very similar between the two thresholds.

### Skill in predicting the forecast distribution

**Fig. 9:**
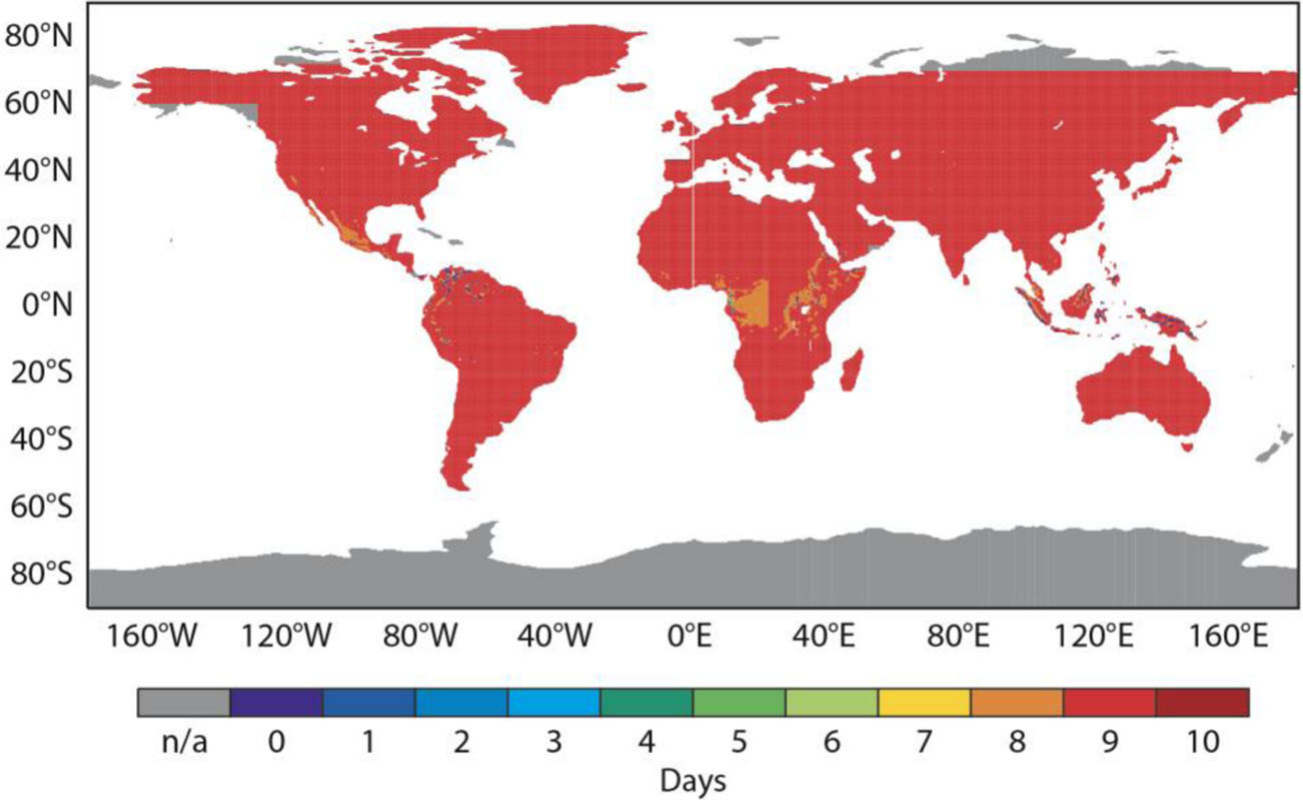
Maximum predictability: forecast lead time at which the CRPSS reaches zero (equivalent to no skill) for the ensemble forecast. The *colour scale* indicates the maximum predictability meaning *dark red* stands for skill full predictability of 10 days

The BSS only evaluates the prediction of exceeding a chosen threshold and not the performance of the forecast over the entire UTCI distribution. This can be achieved using the continuous rank probability skill score (CRPSS). Values of the CRPSS below zero mean that the forecast has no skill. In Fig. 9, the day at which the CRPSS for the ENS probabilistic forecast of UTCI drops to zero is plotted. For example, dark red means that the CRPSS is above zero for the entire forecast range up to 10 days. A predictability of 9 days is achieved for almost all areas of the globe, with the main exception being parts of central Africa, where predictability is slightly lower at 8 days (Fig. 9).

### Case study: Russian heat wave of summer 2010

**Fig. 10:**
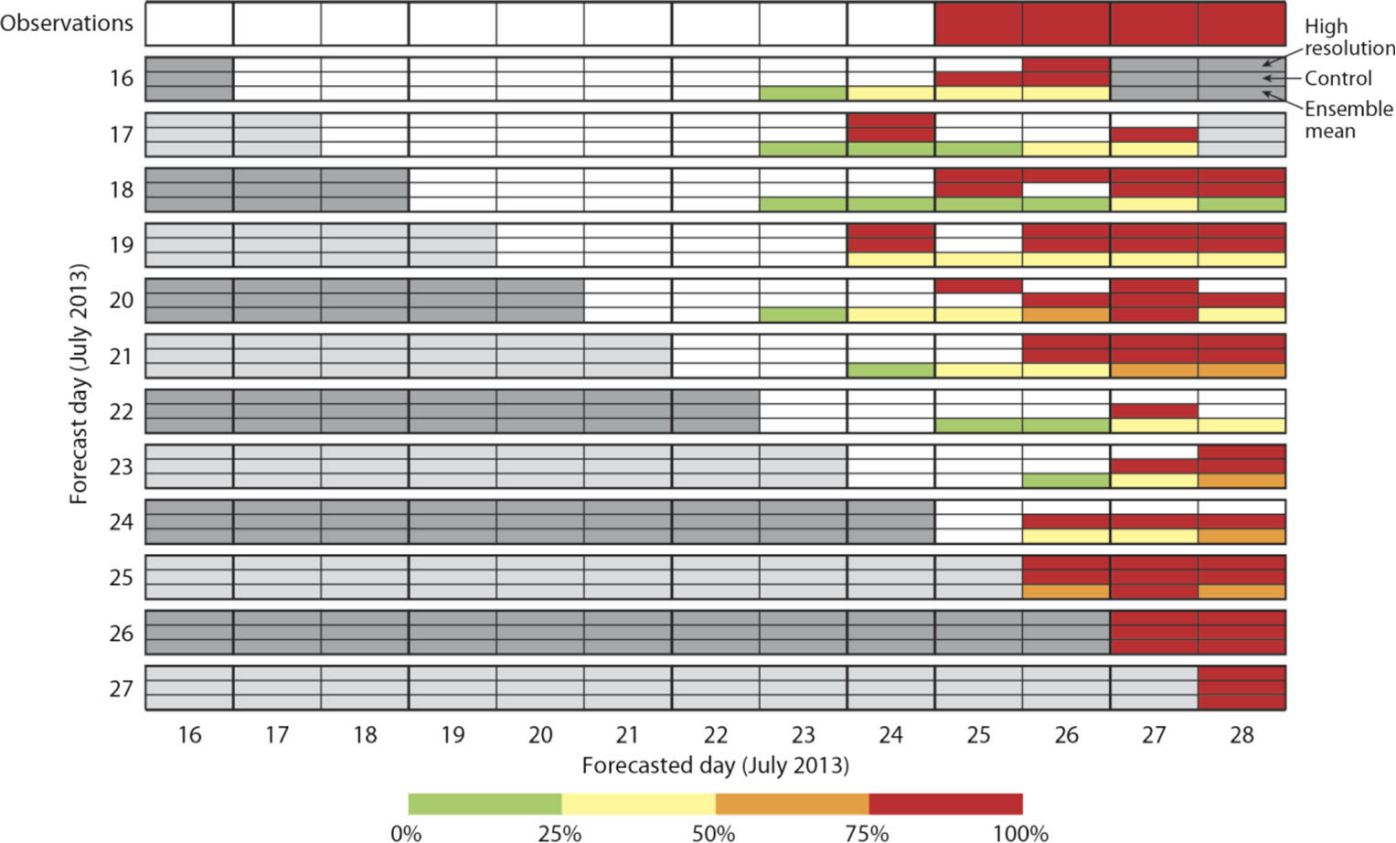
UTCI forecasts for Moscow during the Russian heat wave of summer 2010. The *top line* represents the observations. *Red* indicates days where the UTCI exceeds 32 °C. *All other lines* show forecasts issued on particular days. For example, the *second line* shows a forecast issued on the 16th of July. Each forecast is sub-divided into three sub-boxes. The *top sub*-*box* shows the high resolution (*red* when the forecast UTCI exceeds 32 °C). The *middle sub*-*box* shows the control (*red* when the forecast UTCI exceeds 32 °C). The *bottom sub*-*box* shows the ensemble, and here, the percentage of ensemble members with UTCI exceeding 32 °C is colour coded

In Summer 2010, a blocking anticyclone dominated the weather over Europe and western Russia. This drove cold air towards the Indian Ocean and caused severe flooding in Pakistan as it interacted with warm and humid air. At the same time, warm air from Africa was drawn into western Russia leading to a heat wave with temperatures rising to unprecedented levels (Ghelli et al. 2010). The ECMWF forecasting system demonstrated good predictability for this event. Figure 10 shows a sequence of UTCI forecasts for Moscow where the heat wave started in the last week of July (Ghelli et al. 2010; Katsafados et al. 2013). The top line represents the observations: red indicates days of strong heat stress (UTCI higher than 32 °C). The second row shows the forecasts issued on 16 July; rows below show subsequent forecasts, with the last row showing the forecasts from 27 July. Each forecast row is sub-divided into three sub-boxes. The top sub-box shows the high-resolution forecast (red when the forecast UTCI exceeds 32 °C). The middle sub-box shows the control (red when the forecast UTCI exceeds 32 °C). The bottom sub-box shows the ensemble forecast, and here, the percentage of ensemble members forecasting UTCI to exceed 32 °C is colour coded. From 10 days before the event, the ensemble forecast shows a significant probability for strong heat (for example, the forecast issued on 16 of July is green for the 23 July meaning that up to 25 % of the ensemble members had a UTCI exceeding 32 °C, and yellow for the following days, showing an increase probability of up to 50 %). The signal for the event becomes stronger in the later forecasts: the probabilities for the strong heat stress become progressively higher. The high resolution and control also indicate a possible event, although at the longer range, the timing of this is not consistent from day to day (the signal flip-flops, or changes) (Pappenberger et al. 2011a). These results indicate that a UTCI forecast could have been used to provide an early warning of the onset of this heat wave.

## Conclusion

The skill in forecasting the UTCI has been analyzed for up to 10 days lead time using high-resolution and ensemble NWP forecasts as input. All forecasts have skill in the medium range (up to 10 days); however, the probabilistic UTCI forecasts were the most skilful. The UTCI skill was analyzed globally by comparing the forecast values to UTCI calculated with reanalysis data as a proxy for observation. Although there is a loss in predictability due to the coarse resolution of the global model, the skill demonstrated is very encouraging. It suggests that global probabilistic UTCI predictions can provide a useful global overview of thermal health hazards. The skill of the UTCI predictions shows some regional variations, for example, it is lower in some high altitude areas, and has a strong sensitivity to wind, in particular in coastal regions, which may be related to a maximum wind speed limit under which the UTCI is applicable in this study. Overall, predictability is slightly lower for heat stress than for cold stress. This may be explained by forecast model errors in forecasting of the short- and long-wave radiant fluxes which play a much less important role in the cold.

The skill of the UTCI forecasts depends on the quality of the meteorological input as well as on the construction of the index itself. Here, ECMWF forecasts were used to demonstrate the capability of a current operational global NWP system to predict heat and cold stress using the UTCI. No calibration was applied to the meteorological data to account for model errors, and the resulting UTCI forecast skill can be considered as a benchmark for what may be achieved with calibration or with other models.

The global overviews are not designed to replace higher resolution national forecasts of thermal indices. However, in locations where only simple indices are employed (which do not possess the same degree of thermo-physiological sophistication as the UTCI), this global overview could be of great utility. The evidence provided in this paper suggests that in data sparse regions or when seeking a globally coherent overview of thermal stress risk, the UTCI can be used for disaster preparedness. Considering the global movement of people, the application of a common reference thermal assessment procedure is recommended.

Currently, many countries have implemented Heat Health Warning Systems which have been promoted by the World Meteorological Organization and World Health Organization. These are usually based on simplified thermal assessment procedures, and typically, the national weather services are responsible for the associated warnings. This study shows that UTCI can be applied in daily forecasts and early warnings of extreme weather required for disaster preparedness plans. It also suggests that the use of UTCI may bring added value to current local forecasts in many regions of the world.

## References

[CR1] Bartholmes JC, Thielen J, Ramos MH, Gentilini S (2009). The European Flood Alert System EFAS—part 2: statistical skill assessment of probabilistic and deterministic operational forecasts. Hydrol Earth Syst Sci.

[CR2] Blazejczyk K, Epstein Y, Jendritzky G, Staiger H, Tinz B (2012). Comparison of UTCI to selected thermal indices. Int J Biometeorol.

[CR3] Błażejczyk K, Jendritzky G, Bröde P, Fiala D, Havenith G, Epstein Y, Psikuta A, Kampmann B (2013). An introduction to the Universal Thermal Climate Index (UTCI). Geogr Pol.

[CR4] Broede P, Fiala D, Blazejczyk K, Holmer I, Jendritzky G, Kampmann B, Tinz B, Havenith G (2012). Deriving the operational procedure for the Universal Thermal Climate Index (UTCI). Int J Biometeorol.

[CR5] Cloke HL, Pappenberger F, Renaud JP (2008). Multi-method global sensitivity analysis (MMGSA) for modelling floodplain hydrological processes. Hydrol Process.

[CR6] Courtney J, Lynch P, Sweeney C (2013) High resolution forecasting for wind energy applications using Bayesian model averaging. Tellus A, North America, 65, feb. 2013. Available at http://www.tellusa.net/index.php/tellusa/article/view/19669 Accessed 20 May 2014

[CR7] de Dear RJ, Brager GS (2002). Thermal comfort in naturally ventilated buildings: revisions to ASHRAE Standard 55. Energy Build.

[CR8] Dee DP, Uppala SM, Simmons AJ, Berrisford P, Poli P, Kobayashi S, Andrae U, Balmaseda MA, Balsamo G, Bauer P, Bechtold P, Beljaars ACM, van de Berg L, Bidlot J, Bormann N, Delsol C, Dragani R, Fuentes M, Geer AJ, Haimberger L, Healy SB, Hersbach H, Holm EV, Isaksen L, Kallberg P, Koehler M, Matricardi M, McNally AP, Monge-Sanz BM, Morcrette JJ, Park BK, Peubey C, de Rosnay P, Tavolato C, Thepaut JN, Vitart F (2011). The ERA-Interim reanalysis: configuration and performance of the data assimilation system. Q J R Meteorol Soc.

[CR9] Em-Dat (2014) The OFDA/CRED International Disaster Database –www.emdat.be – Université catholique de Louvain –Brussels – Belgium. Accessed 20 May 2014

[CR10] Fanger PO (1970). Thermal comfort. Analysis and applications in environmental engineering.

[CR11] Fiala D, Lomas KJ, Stohrer M (2001). Computer prediction of human thermoregulatory and temperature responses to a wide range of environmental conditions. Int J Biometeorol.

[CR12] Fiala D, Havenith G, Broede P, Kampmann B, Jendritzky G (2012). UTCI-Fiala multi-node model of human heat transfer and temperature regulation. Int J Biometeorol.

[CR13] Ghelli A, Garcia-Mendez A, Prates F, Dahoui M (2010) Extreme weather events in summer 2010: how did the ECMWF forecasting systems perform? ECMWF Newsletter 125:7–11. Available at www.ecmwf.int Accessed 20 May 2014

[CR14] Giorgi F, Francisco R (2000). Uncertainties in regional climate change prediction: a regional analysis of ensemble simulations with the HADCM2 coupled AOGCM. Climate Dynamics.

[CR15] Hagedorn R, Hamill TM, Whitaker JS (2008). Probabilistic forecast calibration using ECMWF and GFS ensemble reforecasts. Part I: two-meter temperatures. Mon Weather Rev.

[CR16] Hagedorn R, Buizza R, Hamill TM, Leutbecher M, Palmer TN (2012). Comparing TIGGE multimodel forecasts with reforecast-calibrated ECMWF ensemble forecasts. Q J R Meteorol Soc.

[CR17] Hamill TM, Hagedorn R, Whitaker JS (2008). Probabilistic forecast calibration using ECMWF and GFS ensemble reforecasts. Part II: precipitation. Mon Weather Rev.

[CR18] Havenith G (2001). An individual model of human thermoregulation for the simulation of heat stress response. J Appl Physilogy.

[CR19] Havenith G, Fiala D, Blazejczyk K, Richards M, Broede P, Holmer I, Rintamaki H, Benshabat Y, Jendritzky G (2012). The UTCI-clothing model. Int J Biometeorol.

[CR20] Hersbach H (2000). Decomposition of the continuous ranked probability score for ensemble prediction systems. Weather Forecast.

[CR21] Hoeppe P (1999). The physiological equivalent temperature—a universal index for the biometeorological assessment of the thermal environment. Int J Biometeorol.

[CR22] Jendritzky G, Sönning W, Swantes HJ (1979) Ein objektives Bewertungsverfahren zur Beschreibung des thermischen Milieus in der Stadt-und Landschaftsplanung ("Klima-Michel-Modell"). Beitr. Akad. f. Raumforschung u. Landesplanung 28, 85 S

[CR23] Jendritzky G, Havenith G, Weihs P, Batchvarova E (eds) (2009) Towards a Universal Thermal Climate Index (UTCI) for assessing the thermal environment of the human being. Final Report, COST Action Brusseles. Available at http://w3.cost.eu/fileadmin/domain_files/ESSEM/Action_730/final_report/final_report-730.pdf Accessed 20 May 2014

[CR24] Jendritzky G, de Dear R, Havenith G (2012). UTCI—why another thermal index?. Int J Biometeorol.

[CR25] Katsafados P, Papadopoulos A, Varlas G, Papadopoulou E, Mavromatidis E (2013). Seasonal predictability of the 2010 Russian heat wave. Nat Hazards Earth Syst Sci Discuss.

[CR26] Koppe C, Jendritzky G (2005). Inclusion of short-term adaptation to thermal stresses in a heat load warning procedure. Meteorol Z.

[CR27] Koppe C, Kovats S, Jendritzky G, Menne B, Breuer DJ, Wetterdienst D (2004) Heat waves: risks and responses. Regional Office for Europe, World Health Organization

[CR28] Murphy AH (1973). A new vector partition of the probability score. J Appl Meteorol.

[CR29] Pappenberger F, Cloke HL, Persson A, Demeritt D (2011). HESS opinions “On forecast (in)consistency in a hydro-meteorological chain: curse or blessing?”. Hydrol Earth Syst Sci.

[CR30] Pappenberger F, Thielen J, Del Medico M (2011). The impact of weather forecast improvements on large scale hydrology: analysing a decade of forecasts of the European Flood Alert System. Hydrol Process.

[CR31] Parsons KC (2003). Human thermal environments: the effect of hot, moderate and cold environments on human health, comfort and performance.

[CR32] Pickup J, de Dear R (2000) An Outdoor Thermal Comfort Index (OUT_SET*)—part I—the model and its assumptions. In: de Dear R, Kalma J, Oke T, Auliciems A (eds) Conference ICB-ICUC'99 (Sydney, 8-12 Nov. 1999), Biometeorology and Urban Climatology at the Turn of the Millenium, Geneva, 2000. WMO, pp 279-283

[CR33] Pinson P, Hagedorn R (2012). Verification of the ECMWF ensemble forecasts of wind speed against analyses and observations. Meteorol Appl.

[CR34] Pitt M (2008) The Pitt review: learning lessons from the 2007 floods [online]. Available from http://archive.cabinetoffice.gov.uk/pittreview/thepittreview/final_report.html Accessed 20 May 2014

[CR35] Psikuta A, Fiala D, Laschewski G, Jendritzky G, Richards M, Blazejczyk K, Mekjavic I, Rintamaki H, de Dear R, Havenith G (2012). Validation of the Fiala multi-node thermophysiological model for UTCI application. International Journal of Biometeorology.

[CR36] Richardson DS (2000). Skill and relative economic value of the ECMWF ensemble prediction system. Quarterly Journal of the Royal Meteorological Society.

[CR37] Richardson D, Bidlot J, Ferranti L, Haiden T, Hewson T, Janousek M, Prates F, Vitart F (2013) Evaluation of ECMWF forecasts, including 2012-2013 upgrades, ECMWF Technical Memorandum710, ECMWF, Reading, http://www.ecmwf.int/publications/library/ecpublications/_pdf/tm/701-800/tm710.pdf, last accessed: 25.02.2014

[CR38] Roulin E (2007). Skill and relative economic value of medium-range hydrological ensemble predictions. Hydrology and Earth System Sciences.

[CR39] Staiger H, Bucher K, Jendritzky G (1997). Gefühlte Temperatur Die physiologisch gerechte Bewertung von Wärmebelastung und Kältestress beim Aufenthalt im Freien in der Maßzahl Grad Celsius. Annalen der Meteorologie.

[CR40] Staiger H, Laschewski G, Grätz A (2012) The perceived temperature - a versatile index for the assessment of the human thermal environment. Part A: scientific basics. Int J Biometeorol 56:165–176 10.1007/s00484-011-0409-6 21336880

[CR41] Stevenson M (2006). Forecast verification: a practitioner’s guide in atmospheric science. International Journal of Forecasting.

[CR42] VDI (2008). VDI guideline 3787/part 2:environmental meteorology: methods for the human biometeorological evaluation of climate and air quality for urban and regional planning at regional level.

